# Proposal of Allogeochorda gen. nov., Allogeochorda subterranea comb. nov., Allostigmatella gen. nov. and four new combinations and Allosphingorhabdus gen. nov. and Allosphingorhabdus lutea comb. nov. as replacement names for the illegitimate genus names Geochorda, Stigmatella and Alterisphingorhabdus and their species

**DOI:** 10.1099/ijsem.0.007208

**Published:** 2026-06-19

**Authors:** Marko Kostovski, Aharon Oren

**Affiliations:** 1 University Ss Cyril and Methodius, Faculty of Medicine, Institute of Microbiology & Parasitology, Ul. 50-ta Divizija br. 6, 1000 Skopje, North Macedonia; 2 Department of Plant and Environmental Sciences, The Institute of Life Sciences, The Hebrew University of Jerusalem, Edmond J. Safra Campus, Jerusalem 9190401, Israel

**Keywords:** *Geochorda*, *Stigmatella*, *Alterisphingorhabdus*, illegitimate names

## Abstract

The prokaryotic genus names *Geochorda* Karnachuk *et al*. 2025, *Stigmatella* Berkeley and Curtis 1875 (Approved Lists 1980) and *Alterisphingorhabdus* Wang *et al*. 2025 are illegitimate because they are later homonyms of *Geochorda* Cham. & Schltdl. 1828 (a plant genus in the *Plantaginaceae*), *Stigmatella* G. Mey. 1825 (a fungal genus of uncertain position) and *Alterisphingorhabdus* Yan *et al*. 2024 (a bacterial genus in the *Sphingomonadaceae*), respectively, in violation of Rule 51b of the International Code of Nomenclature of Prokaryotes. To resolve these cases of homonymy and to maintain nomenclatural stability, we propose the replacement names *Allogeochorda* gen. nov., *Allogeochorda subterranea* comb. nov. and *Allogeochordaceae* fam. nov.; *Allostigmatella* gen. nov., *Allostigmatella aurantiaca* comb. nov., *Allostigmatella ashevillensis* comb. nov., *Allostigmatella erecta* comb. nov. and *Allostigmatella hybrida* comb. nov.; and *Allosphingorhabdus* gen. nov. and *Allosphingorhabdus lutea* comb. nov., as names replacing the illegitimate ones *Geochorda*, *Geochorda subterranea*, *Geochordaceae*, *Stigmatella aurantiaca*, *Stigmatella ashevillensis* corrig., *Stigmatella erecta*, *Stigmatella hybrida*, *Alterisphingorhabdus* and *Alterisphingorhabdus lutea*, respectively.

## Introduction

The prokaryotic genus names *Geochorda* Karnachuk *et al*. 2025, *Stigmatella* Berkeley and Curtis 1875 (Approved Lists 1980) and *Alterisphingorhabdus* Wang *et al*. 2025 are illegitimate because they are later homonyms of *Geochorda* Cham. & Schltdl. 1828 (a plant genus in the *Plantaginaceae*), *Stigmatella* G. Mey. 1825 (a fungal genus of uncertain position) and *Alterisphingorhabdus* Yan *et al*. 2024 (a bacterial genus in the *Sphingomonadaceae*), respectively. To resolve these cases of homonymy and to maintain nomenclatural stability, we here propose replacement names.

## Status of the name *Geochorda*

The prokaryotic genus *Geochorda* (*Geochordaceae*, *Limnochordales*, *Limnochordia* and *Bacillota*) was described by Karnachuk *et al*. 2025 [1] and was validly published in Validation List 222 [2]. The genus currently contains a single species with a validly published name: *Geochorda subterranea* Karnachuk *et al*. 2025 [1,2]. The generic name *Geochorda* Karnachuk *et al*. 2025 is a homonym of *Geochorda* Chamisso & Schlechtendal 1828, a plant member of the *Plantaginaceae*, *Lamiales*, *Magnoliopsida* and *Tracheophyta* [3]; see also http://www.worldfloraonline.org/taxon/wfo-4000015525, accessed 23 April 2026. Based on Principle 2 and Rule 51b(5) of the International Code of Nomenclature of Prokaryotes (ICNP) [4] and the version in effect at the time of publication [5], the name *Geochorda* proposed for a prokaryotic genus is illegitimate.

The ICNP provides two ways to deal with an illegitimate name: either by introducing a new legitimate replacement name or by submitting a request to the Judicial Commission for an opinion that would permit the illegitimate name to be retained as an exception (see [6,7] for recent examples). As the proposed replacements are unlikely to lead to mistakes or confusion, we choose the first approach. In accordance with Rule 54, we therefore introduce new replacement names that preserve the original meaning of the illegitimate names as initially published.

## Description of *Allo*g*eochorda* Kostovski and Oren gen. nov.

*Allo*g*eochorda* (Al.lo.ge.o.chor’da. Gr. masc. adj. *allos*, other; N.L. fem. n. *Geochorda*, a bacterial genus name; N.L. fem. n. *Allo*g*eochorda*, another *Geochorda*).

Earlier illegitimate homotypic synonym: *Geochorda* Karnachuk *et al*. 2025.

The description of the genus is as given for *Geochorda* Karnachuk *et al*. 2025 [1].

The type species is *Allogeochorda subterranea* (Karnachuk *et al*. 2025) Kostovski and Oren.

## Description of *Allogeochorda subterranea* (Karnachuk *et al.* 2025) Kostovski and Oren comb. nov.

*Allogeochorda subterranea* (sub.ter.ra’ne.a. L. fem. adj. *subterranea*, below the terrestrial ground, referring to the place of isolation).

Illegitimate basonym: *Geochorda subterranea* Karnachuk *et al*. 2025 [2].

The description of the species is as given for *Geochorda subterranea* [1].

The type strain is LN^T^ (=JCM 39508^T^=VKM B-3703^T^), isolated from a deep subsurface aquifer in Chazhemto, Tomsk region, Russia.

The 16S rRNA gene sequence accession number of the type strain is PP660922.

The genome sequence of the type strain can be accessed from GenBank as CP141614.1.

## Description of *Allogeochordaceae* Kostovski and Oren fam. nov.

*Allogeochordaceae* (Al.lo.ge.o.chor.da.ce’ae. N.L. fem. n. *Allogeochorda*, a bacterial genus name; L. fem. pl. n. suff. *-aceae*, ending to denote a family; N.L. fem. pl. n. *Allogeochordaceae*, the *Allogeochorda* family).

The description of the family is as given for the family *Geochordaceae* [1].

The type genus is *Allogeochorda* Kostovski and Oren.

## Status of the name *Stigmatella*

The prokaryotic genus *Stigmatella* (*Archangiaceae*, *Myxococcales*, *Deltaproteobacteria* and *Pseudomonadota*) was described by Berkeley and Curtis 1875 [8,9]. The genus currently contains four species with validly published names: *Stigmatella aurantiaca* Berkeley and Curtis 1875 (Approved Lists 1980) [8,9], *Stigmatella erecta* McCurdy 1971 (Approved Lists 1980) [9,10], *Stigmatella hybrida* Reichenbach 2007 [11,12] and *Stigmatella ashevillensis* corrig. Ahearne *et al*. 2025 [2,13]. The generic name *Stigmatella* Berkeley and Curtis 1875 (Approved Lists 1980) is a homonym of *Stigmatella* G. Mey. 1825 [14] (MycoBank #92371), a fungal member of uncertain position. Under Rule 51b(4) of the ICNP [4], which applies retroactively, the name *Stigmatella* as proposed for a prokaryotic genus is illegitimate. Following Rule 54, we therefore propose replacement names that retain the original meaning of the illegitimate names as first published.

## Description of *Allostigmatella* Kostovski and Oren gen. nov.

*Allostigmatella* (Al.lo.stig.ma.tel’la. Gr. masc. adj. *allos*, other; N.L. fem. n. *Stigmatella*, a bacterial genus name; N.L. fem. n. *Allostigmatella*, another *Stigmatella*).

The description of the genus is as given for *Stigmatella* Berkeley and Curtis 1875 (Approved Lists 1980) [8,9].

The type species is *Allostigmatella aurantiaca* (Berkeley and Curtis 1875) Kostovski and Oren.

## Description of *Allostigmatella ashevillensis* (Ahearne *et al.* 2025) Kostovski and Oren comb. nov.

*Allostigmatella ashevillensis* (a.she.vil.len’sis. N.L. fem. adj. *ashevillensis*, from Asheville, North Carolina, USA, referencing the area of isolation).

Illegitimate basonym: *Stigmatella ashevillensis* corrig. Ahearne *et al*. 2025 [2].

The description of the species is as given for *Stigmatella ashevillensis* corrig. Ahearne *et al*. 2025 [13].

The type strain is NCWAL01^T^ (=ATCC TSD-329=NCCB 100923), isolated from soil in Asheville, North Carolina, USA.

The 16S ribosomal RNA gene sequence is available at GenBank (OP852335.1).

The genome sequence of the type strain can be accessed from NCBI Assembly (ASM2836897v1).

## Description of *Allostigmatella aurantiaca* (Berkeley and Curtis 1875) Kostovski and Oren comb. nov.

*Allostigmatella aurantiaca* (au.ran.ti’a.ca. N.L. fem. adj. *aurantiaca*, orange coloured).

Illegitimate basonym: *Stigmatella aurantiaca* Berkeley and Curtis 1875 (Approved Lists 1980) [9].

The description of the species is as given for *Stigmatella aurantiaca* [8].

The type strain is ATCC 25190 ^T^ (=DSM 17044^T^).

The 16S rRNA gene sequence accession number of the type strain is GU207882.

## Description of *Allostigmatella erecta* (McCurdy 1971) Kostovski and Oren comb. nov.

*Allostigmatella erecta* (e.rec’ta. L. fem. part. adj. *erecta*, erected).

Basonym: ‘*Cystobacter erectus*’ Schroeter 1886 [15]; earlier illegitimate homotypic synonym: *Stigmatella erecta* (Schroeter 1886) McCurdy 1971 (Approved Lists 1980) [10].

The description of the species is as given for *Stigmatella erecta* McCurdy 1971 [10].

The type strain is M26^T^ (=ATCC 25191^T^=DSM 16858^T^), isolated from bark, Canada.

The 16S rRNA gene sequence accession number of the type strain is AJ970180.

## Description of *Allostigmatella hybrida* (Reichenbach 2007) Kostovski and Oren comb. nov.

*Allostigmatella hybrida* (hy’bri.da. L. fem. adj. *hybrida*, half-breed, bastard).

Illegitimate basonym: *Stigmatella hybrida* Reichenbach 2007 [12].

The description of the species is as given for *Stigmatella hybrida* Reichenbach 2007 [11].

The type strain is Sg h20^T^ (=CIP 109130^T^=DSM 14722^T^=JCM 12640^T^), isolated from soil with decaying plant material, Greece.

The 16S rRNA gene sequence accession number of the type strain is DQ768129.

## Status of the name *Alterisphingorhabdus*

The prokaryotic genus *Alterisphingorhabdus* (*Sphingomonadaceae*, *Sphingomonadales*, *Alphaproteobacteria* and *Pseudomonadota*) was described by Wang *et al*. in 2025 [16]. The genus currently contains a single species with a validly published name: *Alterisphingorhabdus lutea* (Baek *et al*. 2019) Wang *et al*. 2025 [16]. The generic name *Alterisphingorhabdus* Wang *et al*. 2025 is a homonym of *Alterisphingorhabdus* Yan *et al*. 2024, a bacterial member of the *Sphingomonadaceae*, *Sphingomonadales*, *Alphaproteobacteria *and *Pseudomonadota* [17]. On the basis of Principle 2 and Rule 51b(5) of the ICNP [4,5], which have retroactive application, the genus name *Alterisphingorhabdus* as proposed for a prokaryote Wang *et al*. in 2025 is illegitimate. Consistent with Rule 54, we therefore propose replacement names that conserve the original meaning of the illegitimate names as initially published.

## Description of *Allosphingorhabdus* Kostovski and Oren gen. nov.

*Allosphingorhabdus* (Al.lo.sphin.go.rhab’dus. Gr. masc. adj. *allos*, another, other, different; N.L. fem. n. *Sphingorhabdus*, a bacterial genus name; N.L. fem. n. *Allosphingorhabdus*, another or different *Sphingorhabdus*).

The description of the genus is as given for *Alterisphingorhabdus* Wang *et al*. 2025 [16].

The type species is *Allosphingorhabdus lutea* (Baek *et al*. 2019) Kostovski and Oren.

## Description of *Allosphingorhabdus lutea* (Baek *et al*. 2019) Kostovski and Oren comb. nov.

*Allosphingorhabdus lutea* (lu’te.a. L. fem. adj. *lutea*, golden-yellow).

Basonym: *Sphingorhabdus lutea* Baek *et al*. 2019 [18].

Illegitimate homotypic synonym: *Alterisphingorhabdus lutea* (Baek *et al*. 2019) Wang *et al. 2025* [16].

The description of the species is as given for *Sphingorhabdus lutea* [18].

The type strain is LPB0140^T^ (=JCM 31568^T^=KACC 18891^T^), isolated from sea water, Orukdo Island, Republic of Korea.

The 16S ribosomal RNA gene sequence is available at GenBank (KX066860). The genome sequence of the type strain can be accessed from NCBI Assembly CP018154.

## Abbreviations

ICNP: International Code of Nomenclature of Prokaryotes
